# Primary histiocytic sarcoma arising in the head and neck with predominant spindle cell component

**DOI:** 10.1186/1746-1596-2-7

**Published:** 2007-02-26

**Authors:** Borislav A Alexiev, Charles J Sailey, Shawn A McClure, Robert A Ord, XF Zhao, John C Papadimitriou

**Affiliations:** 1 University of Maryland Medical Center, Department of Pathology, NBW43, 22 S Greene Street, Baltimore, MD 21201, USA; 2 University of Maryland Medical Center, Department of Oral and Maxillofacial Surgery, 650 West Baltimore Street, Baltimore, MD 21201, USA

## Abstract

This is the first case report of Histiocytic Sarcoma (HS) with predominant spindle cell component occurring in the head and neck region of a 41-year-old man. The tumor was composed of sheets of large round to oval cells with pleomorphic vesicular nuclei, prominent nucleoli and abundant eosinophilic cytoplasm. Multinucleated forms, numerous mitoses, and tumor necrosis were also noted. Sheets, fascicles, and whorls of spindle cells with spindled to ovoid vesicular nuclei, small to medium-sized distinct nucleoli, and eosinophilic cytoplasm were frequently observed. Immunohistochemical staining in the tumor cells was positive for CD163, CD68, lysozyme, CD45, and NSE. Focal expression of CD4 and S-100 was also noted. Electron microscopy demonstrated an abundance of lysosomes in the cytoplasm of tumor cells. Chromosome study revealed a 57–80 hyperdiploid [7]/46, XY [13] karyotype, including 3 to 4 copies of various chromosomes. The immunohistochemical and ultrastructural findings confirmed the diagnosis of HS.

## Background

Histiocytic sarcoma (HS) is rare neoplasm characterized by malignant proliferation of cells showing morphologic and immunophenotypic features similar to those of mature tissue histiocytes [1]. Most patients are adults (median age 46 years). Male predilection is found in some studies [1]. About one-third of cases present in lymph nodes, about one-third in skin, and about one-third in a variety of other extranodal sites, most commonly the intestinal tract [1]. Awareness of HS is important, as the tumor closely mimics other lymphoid tissue malignancies in their clinical presentation and morphologic appearance. We present a case of HS of the head and neck which was initially identified only as malignant spindle cell tumor not further classifiable. To our knowledge, a case of HS with predominant spindle cell component has never been reported before. We describe the histologic, immunohistochemical, and ultrastructural features, as well as the cytogenetics of a HS with unusual differentiation.

## Case presentation

**Figure 1:**
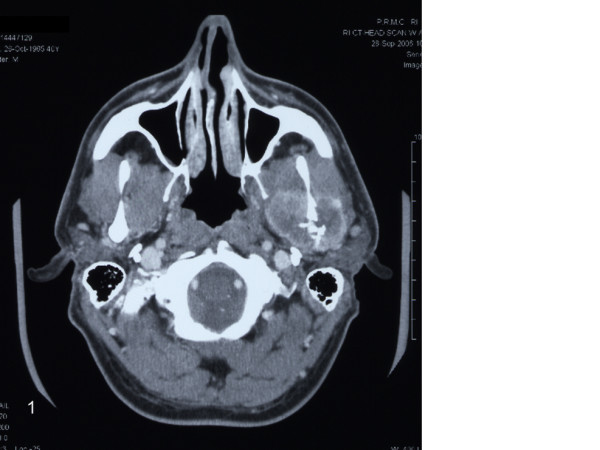
**Histiocytic sarcoma**. Axial CT scan showing a destructive lesion of the left mandible with invasion of the surrounding musculature.

In October of 2006 a 41-year-old otherwise healthy man presented to the University of Maryland, Department of Oral and Maxillofacial Surgery for an evaluation of an expansile mass in the left zygomatic, preauricular region. Five months earlier the patient complained of headaches and increasing fatigue at the end of a normal work day. He then noticed increasing left jaw pain and trismus along with the headaches. He was seen and evaluated by his primary care physician. Initially he was treated for temporomandibular disorder. However, the patient's symptoms failed to subside and subsequently he was referred to an oral and maxillofacial surgeon. Computed tomography of his head and neck was obtained, revealing a destructive mass in the left condyle (Fig. 1). He was subsequently referred to the University of Maryland Medical Center for definitive treatment.

Examination of the patient revealed slight facial asymmetry with a nontender, slightly indurated mass in the left zygomatic, preauricular region. Further examination produced questionable paresthesia in the distribution of the maxillary division of the left trigeminal nerve. No facial nerve weakness was appreciated. Evaluation of the axial and coronal CTs revealed a 4.0 cm soft tissue mass involving the neck of the left condyle, infiltrating the masseter and pterygoid muscles. A whole body PET scan showed increased metabolic activity (SUV 9.2) in the left condyle. No other abnormal activity was seen in the neck, chest, abdomen or pelvis. Laboratory findings: WBC: 4.7 K/mcL, HGB: 13.9 g/dl, HCT: 41.0%, RBC: 4.77 M/mcL, Platelets: 308000 K/mcL. An open biopsy was performed in the operating room via a preauricular incision and a pathological diagnosis of malignant spindle cell tumor was made.

In view of the diagnosis of sarcoma the patient subsequently underwent a vertical compartment resection with exposure via hemicoronal incision extending to a modified Blair incision. The specimen was removed en-bloc with a margin of normal tissue, preserving the facial nerve. The patient was primarily reconstructed with a microvascular free fibula flap from the contralateral leg. He was extubated on post-operative day one, and discharged from the intensive care unit on post-operative day three. The rest of his hospital course was uneventful and he was discharged on post-operative day seven. Following the final pathologic diagnosis he was discussed at the institutional tumor board and recommended for adjunctive radiotherapy.

## Methods

### Gross

Gross examination of the specimen revealed a relatively well circumscribed firm mass (5.6 × 4.2 × 3.2 cm) with yellow-tan cut surface focally infiltrating soft tissue and skeletal muscle, abutting the underlying bone. Areas of necrosis were identified.

### Histology

The resected tissues were fixed in 10% buffered formalin and embedded in paraffin. Subsequently, the tissue blocks were sectioned at a thickness of 5 microns and stained with hematoxylin-eosin.

### Immunohistochemistry

Immunohistochemical staining was performed using Ventana Enhanced DAB Detection Kit and Biotin-StreptAvidin (B-SA) amplified methodology (Ventana, Tucson, AZ) and commercially available prediluted monoclonal antibodies against the following antigens: CD163 (NeoMarkers), CD4 (Biocare Medical), lysozyme, CD1a, CD3, CD8, CD20, CD21, CD23, CD30, CD43, CD45, CD68, CD99, CD117, ALK, S-100 protein, neuron specific enolase (NSE), smooth muscle actin, desmin, vimentin, myogenin, EMA, LMP-1, HMB45, Mart-1/Melan A, TTF1, pancytokeratin, CK 903, CAM 5.2, and Ki-67 (all Ventana, Tucson, AZ).

### In situ hybridization for detection of Epstein-Barr virus

Epstein-Barr (EBV) infection status was analyzed by in situ hybridization for EBV-encoded RNAs using an Epstein-Barr Early RNA Probe Reagent (EBER 1–2, Ventana INFORM EBER, Tucson, AZ).

### Electron microscopy

Representative tissue samples (1 mm cubes) were fixed in 4F1G for 4 hours, postfixed in osmium tetroxide, dehydrated in graded alcohols, and embedded in epoxy resin. The sections were stained with uranyl acetate and lead citrate and examined on a JEM 1200 transmission electron microscope.

### Chromosome study

Cells were dispersed and cultured in RPMI 1640 medium with 20%fetal bovine serum for 24 and 48 hours, respectively. Metaphase cells were analyzed following standard G-banding method. Their karyotypes were interpreted according to the International System for Human Cytogenetic Nomenclature.

## Results

**Figure 2:**
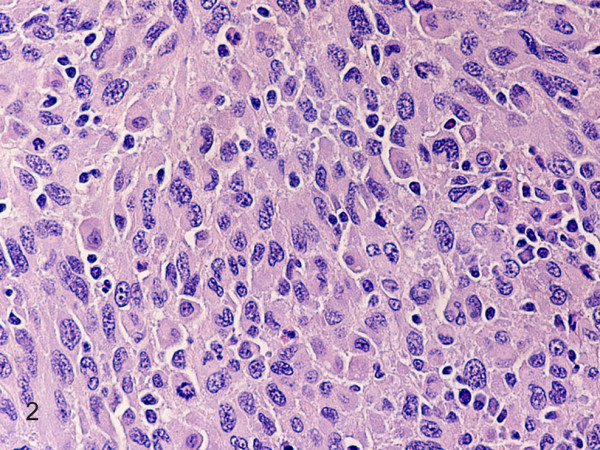
**Histiocytic sarcoma**. Diffuse proliferation of large round to oval cells with pleomorphic vesicular nuclei, prominent nucleoli and abundant eosinophilic cytoplasm. A mitosis is seen. (hematoxylin-eosin, original magnification × 400).

**Figure 3:**
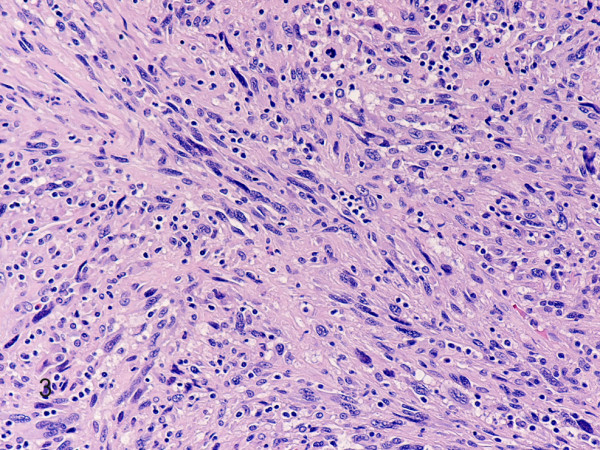
**Histiocytic sarcoma**. Note prominent spindle cell differentiation. (hematoxylin-eosin, original magnification × 200).

**Figure 4:**
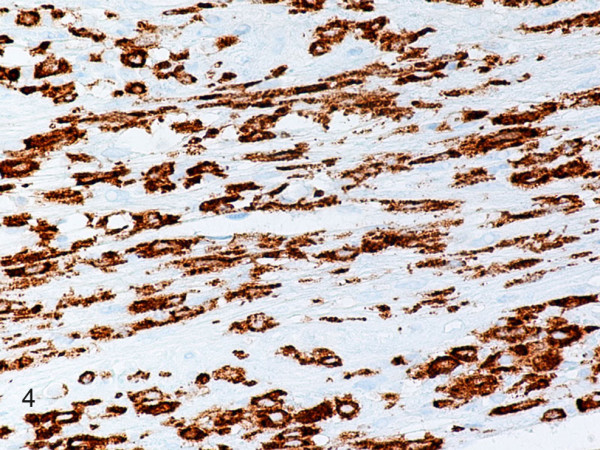
**Histiocytic sarcoma**. Neoplastic round and spindle cells are strongly positive for CD163. (B-SA, anti-CD163, original magnification × 400).

**Figure 5:**
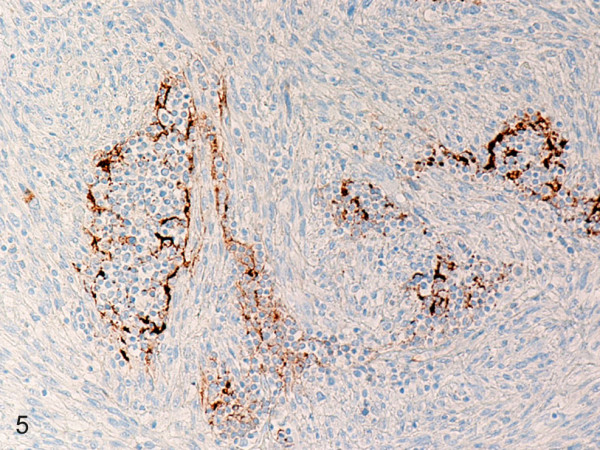
**Histiocytic sarcoma**. Residual follicular dendritic cells are strongly positive for CD21. (B-SA, anti-CD21, original magnification × 200).

**Figure 6:**
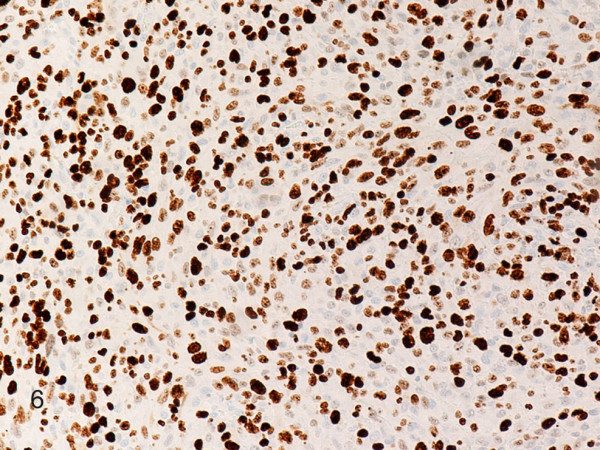
**Histiocytic sarcoma**. Neoplastic cells are strongly positive for Ki-67. (B-SA, anti-Ki-67, original magnification × 200).

**Figure 7:**
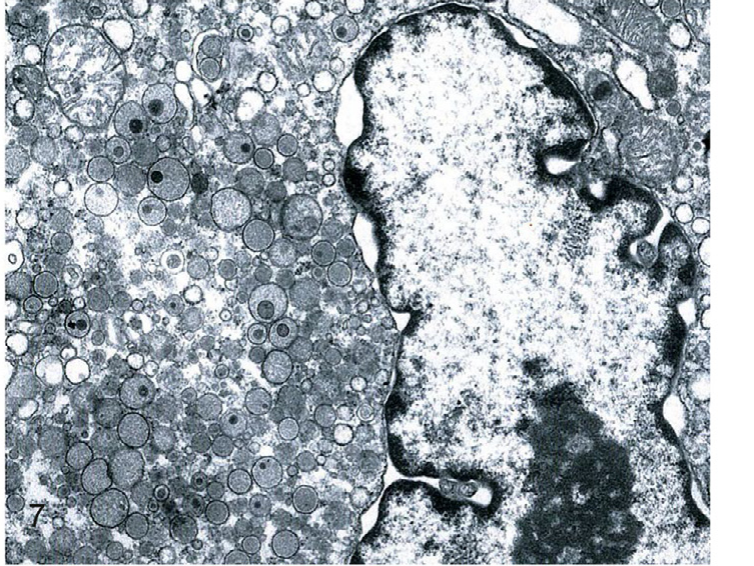
**Histiocytic sarcoma**. Numerous lysosomes are seen in neoplastic cell cytoplasm. (electron microscopy, original magnification × 8000).

Hematoxylin-eosin stained sections showed a tumor mass remotely resembling a completely effaced lymph node, composed of a diffuse proliferation of large round to oval cells with pleomorphic vesicular nuclei, prominent nucleoli and abundant eosinophilic cytoplasm (Fig. 2). Sheets, fascicles, and whorls of spindle cells with spindled to ovoid vesicular nuclei, small to medium, distinct nucleoli, and eosinophilic cytoplasm were frequently observed (Fig. 3). Large multinucleated cells, mitotic figures (27/10 HPFs), and necrosis were also noted. There were numerous admixed lymphocytes, polymorphonuclear leukocytes, and less commonly, plasma cells. The neoplastic cells invaded the lymph node capsule, perinodal soft tissues and periosteum. They were strongly positive for CD163 (Fig. 4), CD68, lysozyme, NSE, and vimentin. Weak to moderate immunostaining for S-100, CD4, and CD45 were also noted. Reactive lymphocytes stained strongly for CD3, CD4, CD8, and to a lesser degree for CD20. Residual CD21 positive, and CD23 positive follicular dentritic cells were also seen (Fig. 5). The Ki-67 index was approximately 70% (Fig. 6). The neoplastic cells were negative for CD43, ALK, CD30, EMA, smooth muscle actin, cytokeratin, desmin, myogenin, TTF1, CD99, CD1a, LMP-1, CD117, HMB45, and Mart-1/Melan A. Electron microscopy revealed presence of numerous lysosomes and lack of desmosomes, Birbeck granules and interdigitating cell processes (Fig. 7). In situ hybridization for EBV-encoded RNAs was negative. Cytogenetic studies revealed a 57–80 hyperdiploid [7]/46, XY [13] karyotype, including 3 to 4 copies of various chromosomes.

## Discussion

Histiocytic and dendritic cell sarcomas are among the rarest of tumors affecting lymphoid tissues [1-5]. These tumors arise from phagocytes and related accessory cells, which have major roles in the processing and presentation of antigens to lymphocytes. Currently, the WHO includes the following 5 entities under this designation: histiocytic sarcoma (HS), follicular dendritic cell sarcoma (FDCS), interdigitating dendritic cell sarcoma (IDCS), Langerhans cell sarcoma (LCS), and dendritic cell sarcoma, not otherwise specified (DCS, NOS)[1]. The pathologic diagnosis of HS is often challenging and requires histologic, immunohistochemical, and, occasionally, electron microscopic analysis [1-5]. We reached the diagnosis of HS in our case based on the exclusion of other sarcomas and carcinomas including other members of the HS/dendritic cell sarcoma group. A comparison between immunophenotypes and ultrastructural features of the neoplastic cells of present case and reported lymphoid tissue phagocytic and accessory cell neoplasms is shown in Table 1.

**Table 1:** Comparison Between Immunophenotypes and Ultrastructural Features of Neoplastic Cells in Present Case and Lymphoid Tissue Phagocytic and Accessory Cells Neoplasms*

IM/US**	**Case**	**HS**	**LCS**	**IDCS**	**FDCS**
Lysozyme	++	++	+	+	+
CD163	++	N/A	N/A	N/A	N/A
CD68	++	++	+	+	+
S-100	+	+	++	++	+
CD1a	-	-	++	-	-
CD21	-	-	-	-	++
CD23	-	-	-	-	++
EMA	-	-	-	-	+
Fascin	N/A	-	-	-	+
Clusterin	N/A	-	-	-	++
CD45	+	+	+	+	++
CD3	-	-	-	-	-
CD4	+	+	+	-	-
CD20	-	-	-	-	-
MHC II	N/A	+	++	+	-
Lysosomes	Numerous	Numerous	Variable	Scattered	Scattered
Birbeck granules	-	-	++	-	-
Desmosomes	-	-	-	-	++
IDCP***	-	-	-	++	-

* References: 1, 5, 6, 10–12

** IM/US: Immunohistochemical marker/ultrastructure

***IDCP: Interdigitating cell processes

+: weak

++: strong

HS: Histiocytic sarcoma

LCS: Langerhans cell sarcoma

IDCS: Interdigitating dendritic cell sarcoma

FDCS: Follicular dendritic cell sarcoma

Histologically, the tumor showed diffuse infiltrate of large, round to ovoid pleomorphic cells; large multinucleated forms, mitoses, and necrosis were commonly seen. Interestingly, our case showed an uncommon phenotype, namely a prominent spindle cell component, causing significant diagnostic confusion with other spindle cell neoplasms. The neoplastic cells demonstrated immunohistochemical staining characteristics similar to those of normal monocytes/histiocytes, namely strong immunoreactivity with CD163, CD68, lysozyme, NSE, and vimentin. In addition, there was focal expression of S-100 protein, CD45, and CD4, reflecting the physiologic pattern of expression of this T-helper antigen by histiocytes [1]. Electron microscopy revealed presence of numerous lysosomes and lack of desmosomes, Birbeck granules and prominent interdigitating cell processes.

Histiocytic sarcoma with a prominent spindle cell component has morphologic similarity with IDCS. The latter consistently expresses S-100 protein, ATPase and HLA-DR, and is variably, weakly positive for CD68, lysozyme, and CD45 [1,5-8]. Unlike HS, IDCS cells do not express non-specific esterases [1]. In addition, cytologic atypia and mitotic rate are low, and necrosis is usually absent [1]. On electron microscopy, IDDCS cells show long cytoplasmic finger-like projections and lack the abundance of lysosomes – a characteristic ultrastructural feature of histiocytes.

In the case presented here, considering the morphologic features, the neoplasm should also be differentiated from a LCS. The neoplastic cells of LCS display overtly malignant cytology and linear nuclear grooves reminescent of Langerhans cell histiocytosis, a key feature to suggest this diagnosis [1]. The neoplastic cells consistently express S-100 protein, and, unlike HS, CD1a [1,5,9]. In addition, there is usually some immunostaining for CD68, lysozyme, and CD45. On electron microscopy, Birbeck granules and a variable number of lysosomes should theoretically be present in all cases in which adequate examination was carried out [1].

Spindle to ovoid cell proliferation with occasional multinucleated cells can be observed in FDCS [1,5,10-12]. The neoplasm forms fascicles, storiform patterns, and whorls [1]. The neoplastic cells are strongly positive for one or more of the follicular dendritic cell markers, including CD21, CD23, and CD35. In addition, they express vimentin, fascin, clusterin, HLA-DR, and are variably positive for EMA, S-100 protein, and CD68 [1,11]. Follicular dendritic cell sarcoma may occur in association with Castleman disease, usually the hyaline vascular type [1]. A high proportion of cases of putative FDCS showing features of inflammatory pseudotumor have been associated with the Epstein-Barr virus [1,12]. In these cases, Epstein-Barr virus encoded RNA (EBER) has been found in all of the spindle cells [1]. In the case presented here, all neoplastic cells were negative for Epstein-Barr encoded RNA. Electron microscopically, the most distinctive ultrastructural feature of FDCS is the presence of numerous long, slender cytoplasmic processes connected by desmosomes.

Dendritic cell sarcoma, NOS, can mimic a HS with prominent spindle cell component. This is a diagnosis of exclusion, not well characterized morphologically and immunohistochemically. The neoplastic cells express CD1a and S-100 protein but lack cytoplasmic Birbeck granules [1].

The overall appearance of HS may be indistinguishable from a diffuse large B-cell lymphoma or an anaplastic large cell lymphoma. Immunohistochemical markers are necessary to make a certain lineage distinction. In contrast to HS, diffuse large B-cell lymphoma constantly express various pan-B markers such as CD19, CD20, CD22, and CD79a, while anaplastic large cell lymphomas are positive for CD30, and ALK [1].

Other tumors, such as carcinomas, melanomas, and soft tissue sarcomas, primary or metastatic, can be confused with HS and were excluded in our case by the lack of immunoreactivity for pancytokeratin, CAM 5.2, CK 903, TTF1, HMB45, MART-1/Melan A, CD99, CD117, smooth muscle actin, desmin, and myogenin.

Last but not least, HS may be confused with reticulohistiocytoma – an uncommon, incompletely characterized benign histiocytic proliferation of the skin and soft tissues. The lesion is composed of epithelioid histiocytes with abundant, densely eosinophilic cytoplasm, and, in contrast to HS, mild if any, nuclear atypia and low mitotic activity [13].

Histiocytic sarcoma is a neoplasm with uncertain molecular pathogenesis. The neoplasm in our case showed a 57–80 hyperdiploid [7]/46, XY [13] karyotype, including 3 to 4 copies of various chromosomes. Recent studies established a cooperative role of PTEN and p16(INK4A)/p14(ARF) in the development of HS [4]. In addition, HS demonstrated germ-like clonal immunoglobulin and T-cell receptor genes [14].

The biologic behavior of HS is typically aggressive with a poor response to therapy. In agreement with previous reports, the HS case presented here demonstrated high proliferative rate and extranodal spread [1]. Stage of disease and possibly tumor size are considered significant prognostic indicators [1]. Most patients die of progressive disease reflecting the high clinical stage at presentation [1]. Sarcomas in the head and neck are best treated initially with surgery to obtain wide surgical margins. This may be limited due to neurovascular structures within the head and neck. Hence local recurrence in large sarcomas is a concern. Radiotherapy has been shown been shown to be an important adjunctive role in management of tumors were wide surgical margins are not possible. Chemotherapy regimens are more controversial. Due to the rarity of head and neck sarcomas there are no proven regiments. Most studies are retrospective reviews from different institutions. Chemotherapy like radiotherapy is used primarily for local control of disease. The patient's overall prognosis is influenced by grade and the ability to obtain wide margins, with improved survivability by controlling local recurrence and distant metastasis [15].

In summary, HS is a rare neoplasm that may pose difficulty in pathologic diagnosis. Awarness of HS is important because these neoplasms may mimic other lymphoproliferative disorders in their clinical presentation and morphologic appearance. Even with immunohistochemical work-up, diagnosis may be missed as histiocytic markers are often not included in the routine pannel of antibodies used for investigation of spindle cell and undifferentiated neoplasms. The key feature suggestive of HS is the strong expression of one or more "histiocytic markers", including CD163, CD68, and lysozyme in the majority of neoplastic cells.

Although a few cases of extranodal FDCS have been described in the head and neck [12], to our knowledge this is the first case of a HS arising in this area.

## Competing interests

The author(s) declare that they have no competing interests.

## Authors' contributions

BAA and CJS processed the specimen, evaluated the immunohistochemical stains, confirmed the diagnosis, designed the report and drafted the manuscript.

SAM and RAO provided surgical intervention and relevant information.

XFZ and JCP provided consultation.

All authors read and approved the final manuscript.
